# Partial inactivation of songbird auditory cortex impairs both tempo and pitch discrimination

**DOI:** 10.1186/s13041-023-01039-5

**Published:** 2023-06-03

**Authors:** Gunsoo Kim, Miguel Sánchez-Valpuesta, Mimi H. Kao

**Affiliations:** 1 Sensory and Motor Systems Research Group, Korea Brain Research Institute, Daegu, South Korea; 2 Department of Biology, Tufts University, Medford, MA 02155 USA; 3 Graduate School of Biomedical Sciences, Tufts University School of Medicine, Boston, MA 02111 USA

**Keywords:** Auditory perception, Auditory processing, Spectrotemporal receptive fields, Songbirds, Operant behavior

## Abstract

Neuronal tuning for spectral and temporal features has been studied extensively in the auditory system. In the auditory cortex, diverse combinations of spectral and temporal tuning have been found, but how specific feature tuning contributes to the perception of complex sounds remains unclear. Neurons in the avian auditory cortex are spatially organized in terms of spectral or temporal tuning widths, providing an opportunity for investigating the link between auditory tuning and perception. Here, using naturalistic conspecific vocalizations, we asked whether subregions of the auditory cortex that are tuned for broadband sounds are more important for discriminating tempo than pitch, due to the lower frequency selectivity. We found that bilateral inactivation of the broadband region impairs performance on both tempo and pitch discrimination. Our results do not support the hypothesis that the lateral, more broadband subregion of the songbird auditory cortex contributes more to processing temporal than spectral information.

**Supplementary Information:**

The online version contains supplementary material available at 10.1186/s13041-023-01039-5.

Spectrotemporal receptive fields (STRFs) describe fundamental tuning properties of auditory neurons. Diverse shapes of STRFs have been observed in cortical neurons across different species [1, 2]. It has been hypothesized that the different types of STRFs underlie basic sound percepts such as pitch and tempo [3]. For example, broadband neurons may primarily extract temporal changes in sound intensity, whereas narrow band neurons may be better at extracting spectral frequency information. However, whether a specific type of tuning contributes to the perception of complex sounds, including learned vocalizations, remains to be tested. Combining perceptual tests with manipulation of neurons with a particular STRF type may allow us to determine how differently-tuned neurons contribute to the perception of a complex sound.

In the songbird auditory cortex field L, neurons are tuned for spectrotemporal modulations of learned songs [4] and receptive fields have been categorized into functional groups based on spectral and temporal tuning properties [3, 5, 6]. Moreover, STRFs are anatomically organized in field L, with spectral tuning widths broadening laterally along the mediolateral axis, and temporal tuning widths broadening along the dorsoventral axis from input to output layers [7]. In the thalamic input-receiving layer, a gradient of spectral tuning widths exist, in which spectral tuning broadens laterally, while temporal tuning remains narrow. The spectral broadening is also observed in the output layers despite a sharp increase in temporal tuning widths. Therefore, the lateral subregion, being less selective for specific frequencies across layers, may be more important for processing temporal information than spectral information. Prior studies have shown that songbirds pay close attention to conspecific vocalizations, making them an excellent model for behavioral investigations of auditory perception [8–10]. As a first step towards linking specific tuning to perception, we pharmacologically inactivated the lateral part of the auditory cortex while songbirds performed auditory discrimination tasks.

Using a two-alternative forced choice task, adult female zebra finches, who do not sing but do discriminate between and show preferences for particular songs and calls [8–11], were trained to discriminate modified zebra finch songs based on tempo or pitch (4 birds in the tempo group and 4 in the pitch group). In the tempo task, five renditions of a conspecific song were sped up or slowed down (± 16%, ± 8%, ± 4%), while maintaining the original pitch. In the pitch task, five renditions of a different conspecific song were shifted up or down in overall pitch (± 1/12, ± 1/24, ± 1/60 octaves), while maintaining the original tempo (Fig. 1A, B; [8]). All stimuli were modified songs and presented in a pseudorandom manner. The tempo group was trained to categorize a song as fast or slow by pressing one of the two response perches, and the pitch group was trained to categorize a song as high or low. Birds were rewarded with food in correct trials and punished in incorrect trials by a lights out period during which all perches stopped working temporarily.

**Fig. 1:**
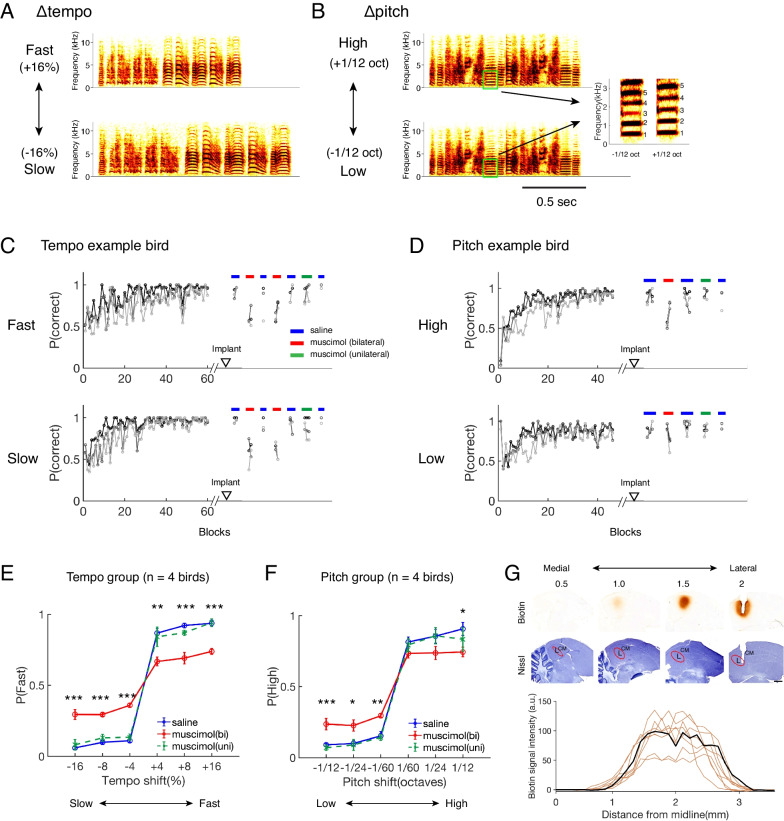
Bilateral muscimol infusion in lateral field L lowers performance on both tempo and pitch discrimination tasks. **A, B** Example song stimuli for tempo (**A**) and pitch (**B**) discrimination tasks. Only the stimuli with largest tempo (± 16%) and pitch shifts (± 1/12 octaves) are shown. Inset in **B** Spectrogram of an example syllable (green boxes) to illustrate pitch shifts (numbers 1 to 5 label corresponding harmonics between the two shifts). **C, D** Example learning curves for one bird trained on the tempo task (**C**) and a different bird trained on the pitch task (**D**). Data are grouped into blocks of 200 trials (~ 3 blocks / day). The lines with different shades of gray (black, dark gray, and light gray) represent 3 different shifts in tempo or pitch with lighter lines representing smaller shifts. The triangles on the x-axis indicate the time of implant surgery. The horizontal bars indicate infusion sessions that occurred on different days (blue: bilateral PBS; red: bilateral muscimol; green: unilateral muscimol). **E, F** Psychometric curves for different infusion sessions (blue: PBS; red: bilateral muscimol; green: unilateral muscimol; mean ± SEM). *p < 0.05, **p < 0.01, ***p < 0.001, Tukey–Kramer post-hoc test (PBS vs. bilateral muscimol). No significant difference found between PBS and unilateral muscimol condition. **G** Photomicrographs of auditory forebrain regions in parasagittal sections that show the extent of biotinylated muscimol spread from ~ 0.5 mm lateral, where no biotin staining is visible, to ~ 2 mm lateral, where a dialysis probe was implanted. Top rows: 40 μm sections stained for biotinylated muscimol and adjacent Nissl stained sections. Bottom plot: the intensity of biotin staining as a function of distance from the midline (1 hemisphere from a tempo bird; 8 hemispheres from 4 pitch birds). The thick black line is from the example sections shown above. L: field L; CM: caudal mesopallium. Scale bar = 1 mm

Birds learned the tasks over a period of weeks (Fig. 1C, D), reaching a performance plateau (> 70% correct responses) first with the stimuli with larger shifts and later with stimuli with smaller shifts (tempo task (mean ± SEM days): 17.5 ± 0.5 days on 16% shifts vs. 34.8 ± 5.5 days on 4% shifts, n = 4 birds; pitch task: 12.0 ± 2.7 days on 1/12octave shifts vs. 22.5 ± 3.6 days on 1/60 octave shifts, n = 4 birds). Once they became proficient at the tasks, performance was maintained at > 70% for all tempo and pitch shifts (Additional file 2: Fig. S1). Mean response latencies measured from the stimulus offset were 0.65 ± 0.06 s for the tempo group and 0.76 ± 0.1 s for the pitch group (p = 0.56, two sample t test).

After each bird learned to discriminate songs based on pitch or tempo, cannulae were implanted and microdialysis probes were inserted in the lateral subregion of field L of both hemispheres to manipulate the activity of neurons with broadband spectral tuning widths [12]. We examined the effects of inactivating the cortical subregion with broader spectral tuning [7] on tempo discrimination and pitch discrimination by reverse-dialyzing muscimol, a potent GABA_A_ receptor agonist (1 mM). Muscimol can inactivate neurons near the infusion site without affecting the fibers of passage and has been used successfully in songbirds [12, 13]. Our histology suggests that the muscimol infusion was concentrated in lateral field L, spanning approximately 1–3 mm lateral to the midline, sparing a substantial medial portion of field L of the infused solutions (Fig. 1G; see Additional file 1: Methods).

Birds’ performance across different conditions was quantified as the probability of responding “fast” or “high” for each tempo or pitch shift (Fig. 1E and F). In the tempo group, during bilateral infusion of phosphate buffered saline (PBS; 0.025 M), performance did not differ from pre-surgery sessions (Fig. 1C and E; p values > 0.8 (pre-surgery vs. PBS) for all tempo shifts, Tukey–Kramer post hoc tests, following ANOVA with pre-surgery, PBS, and muscimol conditions). During bilateral muscimol infusion, performance on tempo discrimination was significantly reduced compared to the PBS condition (from 91 ± 1% to 69 ± 2%, n = 4; Fig. 1C and E; p < 0.0005 for all shifts (PBS vs. bilateral muscimol), Tukey–Kramer post-hoc tests corrected for multiple comparison; see Additional file 2: Fig. S2A for individual performance), although it did remain above chance. Performance recovered upon switching back to PBS. These results indicate that the transient inactivation of the broadband, lateral subregion of field L can impair birds’ ability to discriminate song stimuli based on their tempo.

If spectrally broad lateral subregions are specialized for tempo, inactivating these regions should not disrupt pitch discrimination. During bilateral muscimol infusion in the lateral subregion, however, the pitch group’s overall performance also dropped significantly [from 87 ± 2% to 74 ± 1%; n = 4; Fig. 1D and F; p < 0.05 for − 1/12, − 1/24, − 1/60, and + 1/12 (PBS vs. bilateral muscimol)], although it still remained above chance. The reduction in performance was smaller in the pitch group compared to the tempo group, and for some pitch shifts, performance during muscimol was not significantly different from that during PBS infusion (+ 1/60 oct (p = 0.25) and + 1/24 oct (p = 0.17); see Additional file 2: Fig. S2B for individual performance). Nonetheless, the muscimol-induced performance drop in the pitch group does not support the hypothesis that the lateral subregion of field L is specialized for tempo processing.

In both groups, response rates and latencies did not differ significantly across pre-surgery, PBS, and muscimol conditions, indicating the implantation surgery itself did not cause gross impairments (“tempo group”: response rate (mean ± SEM): 81 ± 3% (pre-surgery), 88 ± 4% (PBS), 83 ± 4% (muscimol), F(2,9) = 1.15, p = 0.36, ANOVA; latency (mean ± SEM): 0.65 ± 0.06 s (pre-surgery), 0.59 ± 0.05 s (PBS), 0.64 ± 0.03 s (muscimol), F(2,9) = 0.29, p = 0.92, ANOVA; “pitch group”: response rate: 78 ± 7% (pre-surgery), 76 ± 9% (PBS), 81 ± 3% (muscimol), F(2,9) = 0.13, p = 0.88, ANOVA; latency: 0.76 ± 0.1 s (pre-surgery), 0.69 ± 0.1 s (PBS), 0.62 ± 0.07 s (muscimol), F(2,9) = 0.1, p = 0.91, ANOVA). We also did not observe significant changes in performance during unilateral infusion of muscimol in both groups (Fig. 1C–F; tempo: p > 0.2, n = 3 birds; pitch: p > 0.7, n = 4 birds, Tukey–Kramer post-hoc tests, following ANOVA with pre-surgery, PBS, unilateral and bilateral muscimol conditions).

In this study, we investigated the link between auditory tuning properties and perception of complex sounds. We asked whether the lateral subregion of the songbird primary auditory cortex, which exhibits broadband spectral tuning, contributes specifically to tempo processing, without affecting pitch discrimination. Our results show that reversible bilateral inactivation of lateral field L caused a significant reduction in performance in both tempo and pitch discrimination. Therefore, while prior work has shown that the spectrotemporal receptive fields of neurons in the songbird auditory cortex are spatially organized, our results do not provide strong support for the hypothesis that different subregions of auditory cortex subserve different percepts.

Several limitations affect our ability to make strong conclusions about whether different subregions of field L differentially contribute to basic perceptual qualities of sound. First, we did not perform the converse experiment—transient inactivation of the medial subregion of Field L—to test whether it differentially affects pitch vs. tempo processing. Second, our muscimol infusion in the lateral field L spread dorsoventrally across all layers of Field L and part of the caudal mesopallium (CM), a secondary cortical region, affecting neurons that may play different roles in tempo and pitch processing due to their markedly different temporal tuning properties [7, 14, 15]. Third, we did not perform neural recordings during muscimol infusion to confirm whether muscimol inactivated lateral neurons while sparing the tuning properties of medial neurons. Although the above chance level performance during muscimol infusion and our histological quantification (Fig. 1G) indicate that a substantial medial portion of field L was spared of muscimol, neural recordings would provide more direct examination of the muscimol effect, including potential changes in tuning via local connections. Finally, while a global gradient in the receptive fields of neurons in field L has been observed in songbirds, more recent studies in birds and mammals have found local heterogeneity in response properties of auditory neurons [16, 17], raising the possibility that local heterogeneity in spectral tuning widths may counteract the effect of inactivation based on a global gradient. Future experiments with a better spatial control of neural activity, such as optogenetic manipulations combined with genetic targeting of specific regions, layers, or even cell types [18], could reveal relationships between spatially segregated sound feature encoding in the auditory cortex and the processing of fundamental percepts of complex sounds.

## Supplementary Information

**Additional file 1. **Methods.

**Additional file 2: Figure S1.** Performance comparison across different magnitudes of tempo and pitch shift before implant surgery. (A–B) Probability of correct trials for different tempo (A) or pitch shifts (B) (mean ± SEM). Different shades of gray for bars indicate difficulty and correspond to those in Fig. 1C and D. Performance was not significantly different across different magnitude of shifts for either group (tempo group: F(5,18) = 2, p = 0.13; pitch group: F (5,18) = 2.64, p = 0.059, ANOVA). **Figure S2. **Performance of individual birds during bilateral muscimol infusion. (A–B) Probability of correct trials for different tempo (A) and pitch shifts (B). The blue and red lines show the average (± SEM) P(correct) for saline and bilateral muscimol conditions, respectively (n = 4 birds on tempo task; n = 4 birds on pitch task). The gray lines show P (correct) for individual birds during bilateral muscimol infusions, and the dashed lines indicate the performance of the two example birds shown in Fig. 1C and D, respectively.

## Data Availability

All data and materials are available from the corresponding author upon reasonable request.

## Abbreviations

STRF: Spectrotemporal receptive fields
PBS: Phosphate buffered saline

## References

[1] Eggermont JJ, Johannesma PM, Aertsen AM (1983). Reverse-correlation methods in auditory research. Q Rev Biophys.

[2] Theunissen FE, Sen K, Doupe AJ (2000). Spectral-temporal receptive fields of nonlinear auditory neurons obtained using natural sounds. J Neurosci.

[3] Woolley SMN, Gill PR, Fremouw T, Theunissen FE (2009). Functional groups in the avian auditory system. J Neurosci.

[4] Moore JM, Woolley SMN (2019). Emergent tuning for learned vocalizations in auditory cortex. Nat Neurosci.

[5] Nagel KI, Doupe AJ (2008). Organizing principles of spectro-temporal encoding in the avian primary auditory area field L. Neuron.

[6] Nagel K, Kim G, McLendon H, Doupe A (2011). A bird brain’s view of auditory processing and perception. Hear Res.

[7] Kim G, Doupe A (2011). Organized representation of spectrotemporal features in songbird auditory forebrain. J Neurosci.

[8] Nagel KI, McLendon HM, Doupe AJ (2010). Differential influence of frequency, timing, and intensity cues in a complex acoustic categorization task. J Neurophysiol.

[9] Elie JE, Theunissen FE (2018). Zebra finches identify individuals using vocal signatures unique to each call type. Nat Commun.

[10] Woolley SC, Doupe AJ (2008). Social context-induced song variation affects female behavior and gene expression. PLoS Biol.

[11] Paul A, McLendon H, Rally V, Sakata JT, Woolley SC (2021). Behavioral discrimination and time-series phenotyping of birdsong performance. PLoS Comput Biol.

[12] Moorman S, Ahn J-R, Kao MH (2021). Plasticity of stereotyped birdsong driven by chronic manipulation of cortical-basal ganglia activity. Curr Biol.

[13] Tanaka M, Singh Alvarado J, Murugan M, Mooney R (2016). Focal expression of mutant huntingtin in the songbird basal ganglia disrupts cortico-basal ganglia networks and vocal sequences. Proc Natl Acad Sci.

[14] Lim Y, Lagoy R, Shinn-Cunningham BG, Gardner TJ (2016). Transformation of temporal sequences in the zebra finch auditory system. Elife.

[15] Calabrese A, Woolley SMN (2015). Coding principles of the canonical cortical microcircuit in the avian brain. Proc Natl Acad Sci.

[16] Araki M, Bandi MM, Yazaki-Sugiyama Y (2016). Mind the gap: neural coding of species identity in birdsong prosody. Science.

[17] Zeng H, Huang J, Chen M, Wen Y, Shen Z, Poo M (2019). Local homogeneity of tonotopic organization in the primary auditory cortex of marmosets. Proc Natl Acad Sci.

[18] Lee C, Lavoie A, Liu J, Chen SX, Liu B (2020). Light up the brain: the application of optogenetics in cell-type specific dissection of mouse brain circuits. Front Neural Circuits..

