# Supplementary material for: Partial inactivation of songbird auditory cortex impairs both tempo and pitch discrimination
Source: Mol Brain. 2023 Jun 3;16:48. doi: 10.1186/s13041-023-01039-5 (PMC10239083; doi:10.1186/s13041-023-01039-5)
Supplement: Supplementary file 1 — Additional file 1. Methods. [file 13041_2023_1039_MOESM1_ESM.docx]

**Materials and methods**

All procedures were performed in accordance with protocols approved by the University of California, San Francisco Institutional Animal Care and Use Committee.

*Stimuli*

Songs from two male zebra finches were used for tempo and pitch tasks, respectively. For the tempo task, song renditions were either sped up or slowed down (±16%, ±8%, ±4%), while preserving the overall pitch. This was accomplished by using a phase vocoding algorithm (a Matlab implementation by DPW Ellis at <http://www.ee.columbia.edu/~dpwe/resources/matlab/pvoc/>). Briefly, short time Fourier transform was performed on overlapping segments of the song, and the resulting spectrogram representation was interpolated to compress or stretch in time. This changed the tempo, while preserving the spectral structure [8]. For the pitch task, song renditions from a different male were shifted in overall pitch (±1/12, ±1/24, ±1/60 octaves), while preserving the song tempo. Song renditions were first compressed or stretched in the time domain to shift the pitch, and the spectrogram representations of the modified renditions were then interpolated to the original duration. For the tempo task, 15 stimuli were sped up (5 renditions x 3 tempo shifts) and 15 were slowed down. For the pitch task, 15 stimuli were shifted up in pitch (5 renditions x 3 pitch shifts) and 15 were shifted down. The manipulated songs were played out at the overall RMS level of 70dB SPL.

*Operant behavior*

Adult female zebra finches (> 90 days post hatch) were trained to discriminate tempo- or pitch-shifted songs using a two alternative forced choice paradigm [8]. Female zebra finches do not sing, but they exhibit sophisticated perceptual abilities, readily discriminating individual males based on their songs or calls and showing preferences for particular songs based on social context ([9,10]; Geberzahn and Derégnaucourt, 2020). Female zebra finches have been used in a number of operant conditioning experiments ([8,11]; Benichov et al., 2016; Coleman et al., 2019; Geberzahn and Derégnaucourt, 2020), and show similar learning curves and reaction times as male zebra finches (Rouse et al., 2022). Birds were singly housed, and each cage was equipped with a center perch that started a trial by triggering a song playback (“song perch”), and two response perches. A feeder was raised from below the cage floor so that birds could reach food only when the feeder was up. A custom written program (Matlab or Python) controlled the behavioral apparatus, and recorded the responses (TDT RX8, arduino, or BeagleBone micro-controllers).

Initially, birds learned to hop on the song perch to trigger song playback. Then, birds were required to press the song perch followed by a response perch within 3 seconds after the completion of a playback - one perch for fast (or high pitch) and the other for slow (or low pitch) stimuli. Birds were required to wait until the playback ended before responding. For correct responses, birds received a food reward; for incorrect responses, a 30 s time out during which the lights went out and the perches were not responsive. Birds worked for food rewards, and water was provided *ad libitum*.

*Drug infusion*

Muscimol (1 mM) or PBS (0.025M) was infused into the lateral auditory forebrain using reverse microdialysis. Muscimol is a potent GABA_A_ receptor agonist (EC50 ~100 nM, Bader et al., 2017) and has an advantage for local inactivation since it acts on local neurons without blocking action potential generation in the fibers on passage, unlike sodium channel blockers. It has been used successfully in a number of songbird studies ([12,13]; Stepanek and Doupe, 2010; Tachibana et al., 2022). Once birds’ performance has reached a plateau (> 70% correct responses), cannulae for microdialysis probes (CMA, Sweden) were implanted bilaterally with the tip location targeted for the middle layer of field L (AP = 1.5 mm anterior to the Y sinus; ML = 2 mm; DV = 1.4 mm). Next day, dialysis probes were inserted and pieces of tubing (~5 cm) were connected to the inlets of the probes and functioned as drug reservoir [12], which was filled with PBS or muscimol using a syringe pump (Harvard apparatus) for 5 minutes (15 μl/min). The first set of data with dialysis probes were collected in the presence of PBS (> 500 trials) before it was switched to muscimol (1 mM) the following day. After a bilateral muscimol infusion, PBS was infused to confirm the recovery of performance before additional muscimol (bilateral or unilateral) infusions. At the end of behavioral experiments, biotinylated muscimol was reverse-dialyzed for histological estimation of drug spread [12].

To estimate the extent of muscimol spread, parasagittal sections (40 μm thick) were processed for biotin staining (at 120 μm mediolateral intervals) and neighboring sections for Nissl staining. To quantify biotin staining intensity, biotin-stained slides (1 hemisphere from a tempo bird; 8 hemispheres from 4 pitch birds) were imaged with a slide scanning microscope (Pannoramic Scan II, 3DHistech) ensuring the same imaging conditions for all brain sections. In every section, a pair of 300 μm (or 20 pixels)-wide circular ROIs were drawn, one over the center of the biotin-stained region and another over a background region > 2 mm away (ImageJ, 32-bit grayscale). The mean values for each ROI were obtained using ImageJ’s mean gray value measuring tool, then background values were subtracted in each section. The background subtracted intensity values were aligned by the probe location at ~2 mm and plotted as a function of the distance from the midline (Fig. 1G). Our quantification showed at ~1 mm lateral to the midline (i.e., 1 mm medial to the probe location), biotin intensity dropped to less than 5% of the center values, and at ~0.6 mm lateral to the midline, no biotin staining was detected. Assuming field L extends ~2.5 mm laterally from the midline, we estimate that ~40% of the medial portion of field L (from the midline to ~1 mm laterally) was spared of muscimol.

*Data analysis*

Learning curves were constructed by counting correct and incorrect trials in blocks of 200 trials. Probability of being correct was calculated for each tempo or pitch shift that had 5 exemplars. Behavioral performance was defined as the number of correct trials divided by the sum of correct and incorrect trials. It was determined that a bird has learned the task when performance reached above 70% and became stable. We note that during training, birds’ performance was lower initially for smaller shifts (i.e., more difficult), but with time, they improved significantly on all shifts, and there was no significant difference in performance across varying magnitudes of shift (Fig. S1). Although the birds are most likely to use the most prominent cues, the lack of clear performance change over a range of difficulties in trained birds (before implant surgery) suggest that birds may also use other cues. Performance measure at yet smaller shifts might resolve this issue.

For trials with reverse dialysis, to guard against drug wash out, data were included for analysis only up to the first 5 hours of each drug session, during which the effect of bilateral muscimol was stable (average number of trials during the 5 hour period (mean ± SEM): 1090 ± 537 trials (PBS), 820 ± 141 trials (muscimol), n = 8 birds). PBS or muscimol infusion was performed on different days, and when one condition was repeated on multiple days, the data were pooled across infusions. Statistical significance of performance differences was assessed across pre-surgery, PBS, and muscimol conditions using one way ANOVA followed by Tukey-Kramer post-hoc tests for pairwise comparisons corrected for multiple comparisons unless stated otherwise. In our design, birds were trained to perform either the tempo or pitch task, which did not allow us to compare their performance on tempo and pitch for individual birds.

**Supplemental references**

Geberzahn N, Derégnaucourt S. Individual vocal recognition in zebra finches relies on song syllable structure rather than song syllable order. Journal of Experimental Biology. 2020;223:jeb220087.

Benichov JI, Benezra SE, Vallentin D, Globerson E, Long MA, Tchernichovski O. The Forebrain Song System Mediates Predictive Call Timing in Female and Male Zebra Finches. Current Biology. 2016;26:309–18.

Coleman MJ, Saxon D, Robbins A, Lillie N, Day NF. Operant Conditioning Task to Measure Song Preference in Zebra Finches. J Vis Exp. 2019.

Rouse AA, Patel AD, Wainapel S, Kao MH. Within-species differences in vocal production learning in a songbird are associated with differences in flexible rhythm pattern perception. 2022; <http://biorxiv.org/lookup/doi/10.1101/2022.07.13.499954>

Bader BM, Steder A, Klein AB, Frølund B, Schroeder OHU, Jensen AA. Functional characterization of GABAA receptor-mediated modulation of cortical neuron network activity in microelectrode array recordings. PLOS ONE. 2017;12:e0186147.

Stepanek L, Doupe AJ. Activity in a Cortical-Basal Ganglia Circuit for Song Is Required for Social Context-Dependent Vocal Variability. Journal of Neurophysiology. 2010;104:2474–86.

Tachibana RO, Lee D, Kai K, Kojima S. Performance-Dependent Consolidation of Learned Vocal Changes in Adult Songbirds. J Neurosci. 2022;42:1974–86.
