# Supplementary material for: Partial inactivation of songbird auditory cortex impairs both tempo and pitch discrimination
Source: Mol Brain. 2023 Jun 3;16:48. doi: 10.1186/s13041-023-01039-5 (PMC10239083; doi:10.1186/s13041-023-01039-5)
Supplement: Supplementary file 2 — Additional file 2: Figure S1. Performance comparison across different magnitudes of tempo and pitch shift before implant surgery. (A–B) Probability of correct trials for different tempo (A) or pitch shifts (B) (mean ± SEM). Different shades of gray for bars indicate difficulty and correspond to those in Fig. 1C and D. Performance was not significantly different across different magnitude of shifts for either group (tempo group: F(5,18) = 2, p = 0.13; pitch group: F (5,18) = 2.64, p = 0.059, ANOVA). Figure S2. Performance of individual birds during bilateral muscimol infusion. (A–B) Probability of correct trials for different tempo (A) and pitch shifts (B). The blue and red lines show the average (± SEM) P(correct) for saline and bilateral muscimol conditions, respectively (n = 4 birds on tempo task; n = 4 birds on pitch task). The gray lines show P (correct) for individual birds during bilateral muscimol infusions, and the dashed lines indicate the performance of the two example birds shown in Fig. 1C and D, respectively. [file 13041_2023_1039_MOESM2_ESM.pdf]

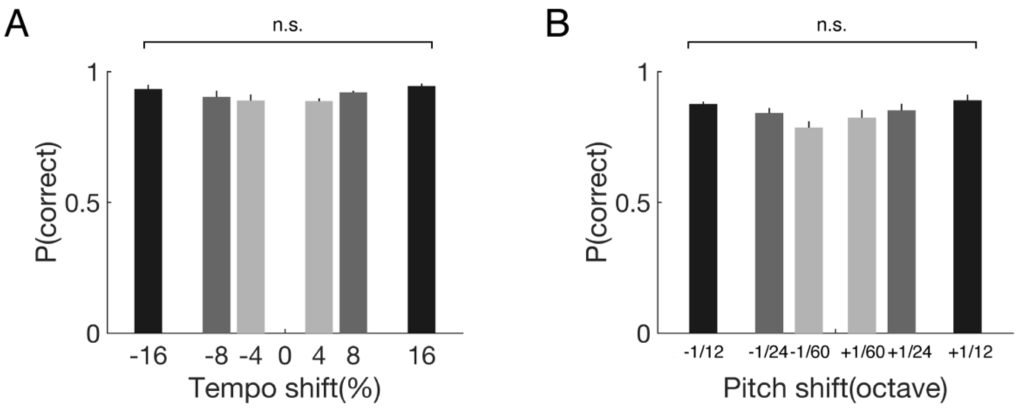

Figure S1. Performance comparison across different magnitudes of tempo and pitch shift before implant surgery. (**A-B**) Probability of correct trials for different tempo (A) or pitch shifts (B) (mean ± SEM). Different shades of gray for bars indicate difficulty and correspond to those in Fig. 1C and 1D. Performance was not significantly different across different magnitude of shifts for either group (tempo group: $F(5,18) = 2$, $p = 0.13$; pitch group: $F(5,18) = 2.64$, $p = 0.059$, ANOVA).

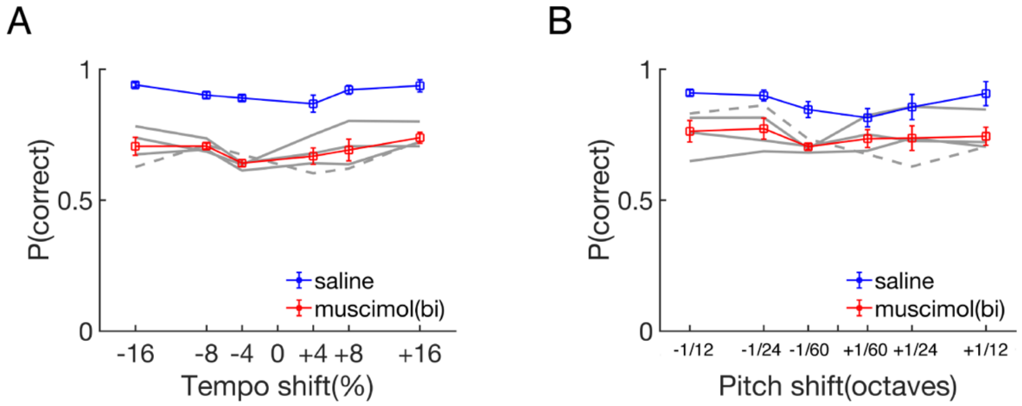

Figure S2. Performance of individual birds during bilateral muscimol infusion. (**A-B**) Probability of correct trials for different tempo (A) and pitch shifts (B). The blue and red lines show the average (±SEM) P(correct) for saline and bilateral muscimol conditions, respectively (n = 4 birds on tempo task; n = 4 birds on pitch task). The gray lines show P(correct) for individual birds during bilateral muscimol infusions, and the dashed lines indicate the performance of the two example birds shown in Fig. 1C and D, respectively.
